# Serious Multiorgan Toxicity Caused by Mixed Herbal Tea Ingestion: A Case Report

**DOI:** 10.7759/cureus.34000

**Published:** 2023-01-20

**Authors:** Raziye Yazıcı, İbrahim Güney

**Affiliations:** 1 Department of Internal Medicine, Division of Nephrology, Konya Beyhekim Training and Research Hospital, Konya, TUR; 2 Department of Internal Medicine, Division of Nephrology, University of Health Sciences, Konya City Hospital, Konya, TUR

**Keywords:** bone marrow toxicity, hepatotoxicity, interstitial nephritis, renal failure, multiorgan toxicity, herbal tea

## Abstract

Recent years have witnessed a growing trend in the use of complementary and alternative herbal products. However, the ingestion of some herbal products may cause a wide spectrum of adverse effects. We report a case of multiorgan toxicity following the ingestion of mixed herbal tea. A 41-year-old woman presented to the nephrology clinic with complaints of nausea, vomiting, vaginal bleeding, and anuria. She had consumed a glass of mixed herbal tea three times a day after meals for three days, to lose weight. Initial clinical and laboratory findings showed serious multiorgan toxicity including hepatotoxicity, bone marrow toxicity, and nephrotoxicity. Although herbal preparations are marketed as natural products, they may cause various toxic effects. There should more efforts to raise public awareness about the possible toxic effects of herbal preparations. Clinicians should consider the ingestion of herbal remedies as an etiology when encountering patients with unexplained organ dysfunctions.

## Introduction

Over the past few years, there has been a growing trend in the use of herbal medicine for various healthcare purposes: to cure or to prevent diseases or to lose weight, etc. [1,2]. Many people assume that herbal products are natural and hence harmless and not toxic. But the efficacy and safety of some herbal supplements or mixtures of them have not been properly tested [3]. Ingestion of some herbal products may cause a wide spectrum of adverse effects ranging from mild allergic or mild gastrointestinal events to life-threatening complications, and even prove to be fatal in some cases [4,5]. These may be due to toxic effects of biological substances in the herbal remedies or harmful contaminants in these preparations, which may be from plant raw materials or from the manufacturing process or caused by the interaction between the herbal product and the drug taken concomitantly or the consumption of more than one herbal product concomitantly [4,5].

To prevent severe risks due to the misuse of herbal products, it is very important to raise awareness among the public and healthcare professionals. Reporting cases that involve severe toxicity resulting from the misuse of herbal products may increase awareness of this issue. In light of this, we report a case of serious multiorgan toxicity including hepatotoxicity, bone marrow toxicity, and nephrotoxicity, following the ingestion of mixed herbal tea in a 41-year-old female.

## Case presentation

A 41-year-old woman presented to the nephrology clinic with complaints of nausea, vomiting, low urine output, and vaginal bleeding. It was informed that two days ago, she had presented to the emergency department with complaints of fatigue, nausea, and widespread pain, and hospitalization had been recommended due to low blood count and deterioration found in liver and kidney function tests at that time. However, the patient had refused hospitalization, further investigation, and treatment. Her past medical history and family history were unremarkable. Before admission to the emergency department, a detailed history-taking revealed that she had drunk a glass of mixed herbal tea three times a day after meals for three days, to lose weight. This mixed herbal tea had been prepared by boiling and brewing the mixture of seven different herbals. This mixture contained linden (Tilia), turmeric (Curcuma longa), ginger (Zingiber officinale), sage tea (Salvia), rosa-sinensis (hibiscus flower), yarrow (Achillea millefolium), and rosehip (Rosa canina). The effects of turmeric (Curcuma longa) and ginger (Zingiber officinale) on weight loss have been described in the literature [6,7]. She had obtained this mixture from a herbalist. We could not ascertain the amount and proportion of each of the seven different herbs in this mixture.

The patient had no fever or diarrhea and the findings of the physical examination were insignificant except for the decreased turgor and tone of the skin and increased bowel sounds. She weighed 93 kg and her body mass index was 35.4 kg/m^2^. Some of the initial laboratory test results were as follows - platelet count: 14,000/µL; serum creatinine level: 8.45 mg/dL; aspartate aminotransferase (AST) level: 131 U/L; alanine aminotransferase (ALT) level: 318 U/L; lactate dehydrogenase (LDH): 3,279 U/L; procalcitonin: 100 ng/mL; C-reactive protein (CRP): 189 mg/L. Laboratory measurements of the patient are summarized in Table 1.

**Table 1:** Laboratory measurements of the patient ALT: alanine aminotransferase; AST: aspartate aminotransferase; WBC: white blood cell; CRP: C-reactive protein

Variables	At the emergency department	First day of hospitalization	Fourth day of hospitalization	20th day of hospitalization	At discharge	Fourth month from hospital admission	First year from hospital admission
Urea, mg/dL (normal range: 6-20)	41	169	107	95	101	62	15
Creatinin, mg/dL (normal range: 0.5-0.9)	1.8	8.45	7.95	6.27	3.46	2.45	1.18
Albumin, g/dL (normal range: 3.5-5.2)		3	2.5		3.8	3.8	4
AST, U/L (normal range: 0-32)	1,164	131	19	10	8		15
ALT, U/L (normal range: 0-33)	655	318	49	4	12	15	12
WBC count/mL (normal range: 4,500-10,000)	2,010	5,200	7,010	5,170	11,000	5,540	7,110
Hemoglobin, g/dL (normal range: 12-16)	14.5	11.5	7.7	9.3	10.9	10.6	12.7
Platelet count/µL (normal range: 150-450)	73,000	14,000	59,000	166,000	187,000	162,000	185,000
Procalcitonin, ng/mL (normal range: 0-0.1)		100	73	0.2			
CRP, mg/L (normal range: 0-5)	31	189	47	5	3		
Urine output, mL/day	300	0	50	2,300	3,400	2,000	2,000

A blood smear examination showed only a finding of thrombocytopenia, and there was no finding of hemolysis. Due to a lack of urine output, a urine analysis could not be performed. HBsAg, Anti-HCV, Anti-HIV, and Anti-HAV IgM screening were all negative. There was no remarkable finding on chest X-ray and abdominal ultrasonography, except for the increased echogenicity of the kidneys. The patient was hospitalized and intravenous fluid infusion was initiated. After blood samples for culture were taken, she was started on meropenem 500 mg/day empirically. The patient received a platelet transfusion and underwent hemodialysis due to anuria, deterioration of kidney functions, and uremic symptoms. Hemoglobin level decreased rapidly and a red blood cell transfusion was given. A gynecological consultation for severe vaginal bleeding gave no recommendation except for platelet and red blood cell transfusion and follow-up. On the fourth day of hospitalization, platelet count increased to 60,000/µL, and AST and ALT decreased to 19 and 49 U/L respectively. The amount of vaginal bleeding also decreased, but urine output and renal functions did not improve. The results of anti-neutrophil cytoplasmic antibody (ANCA) and anti-nuclear antibody (ANA) tests were negative. Blood culture results were negative for any infectious diseases. Diagnostic renal biopsy could not be performed due to thrombocytopenia initially and due to patient refusal after thrombocytopenia improved. Acute nephritis due to herbal tea ingestion was considered, and the patient was started on 1 mg/kg/day of methylprednisolone on the 12th day of hospitalization. After that, urinary output increased and kidney functions improved gradually within days. She was treated with methylprednisolone for 10 consecutive days, which was tapered over a period of eight weeks. She was discharged from the hospital on the 40th day of hospitalization and educated about the possible adverse effects of herbal preparations.

At a follow-up from the fourth month of hospitalization, there was no finding of hematuria or proteinuria in urine analysis; serum creatinine and urea levels were 2.45 mg/dl and 62 mg/dl, respectively. After one year, the serum creatinine level of the patient was found to be 1.18 mg/dl (Table 1).

## Discussion

There are so many unknown issues about herbal preparations, such as their safety/adverse effects, their interactions with other herbs or drugs used concomitantly, etc. [1,8]. Most of the information about these toxic effects has been provided by case reports, and hence it is difficult to establish a causal relationship. The actual incidence of adverse effects of herbal remedies is not known. In a systematic review by Lee et al., the incidence of adverse events related to herbal drugs was 3.1%, and the most frequently reported adverse events were digestive symptoms (44.3%) followed by nervous system-related symptoms (17.3%) [8].

Approximately 20% of acute kidney injury cases are xenobiotic-induced [9]. This kind of injury may occur due to direct and/or immune-mediated nephrotoxic effects including direct tubule effects and interstitial nephritis. In herb-induced acute interstitial nephritis, renal tubular epithelial cells are the main targets, and progression to a chronic state may occur. Aristolochic acid (AA) nephropathy is the best-known herb-induced chronic kidney disease associated with progressive interstitial nephritis [10].

In our case, it was considered that bone marrow depletion, liver dysfunction, and acute renal failure were caused by mixed herbal tea ingestion, and there was no other factor that might have led to this multiorgan dysfunction. There was no drug history, no gastroenteritis, or no finding of hemolysis or infection, except for increased procalcitonin and CRP levels; blood and urine culture results were negative, and although bone marrow depletion and liver dysfunction improved in a short time, renal dysfunction persisted for a longer period of time; hence, this situation could not be explained with infection. Renal dysfunction following herbal tea ingestion may be a consequence of acute interstitial nephritis or acute tubular necrosis. Long-lasting renal dysfunction, in our case, indicated the probability of interstitial nephritis. Renal biopsy could not be performed, initially due to thrombocytopenia and later due to patient refusal.

The Naranjo nomogram for adverse drug reaction assessment is used for determining the likelihood of whether an adverse event is actually due to the drug or not [11]. To validate our perception that the multiorgan dysfunction of our patient was due to herbal tea ingestion rather than other factors, we applied the Naranjo nomogram to our case, and the score obtained in our case was 7, which placed it in the probable range.

Usually, increased procalcitonin and CRP levels bring to mind bacterial infection and sepsis; however, several previous studies have reported that procalcitonin levels might increase in the absence of infectious diseases [12-14]. In a study involving patients who underwent cardiac surgery, it was concluded that in patients with acute kidney injury, high procalcitonin level was a marker of acute kidney injury and would not be able to differentiate infected from non-infected patients [13]. In another study, an extremely high serum procalcitonin level was detected in a patient with serious amphetamine intoxication but without bacterial infection [14]. In our patient, the increase in procalcitonin level may have been caused by plant toxins. Rapid improvement after hemodialysis can be attributed to the removal of these toxins via hemodialysis. Our patient had taken a mixture of herbs of unknown proportions and amounts, and hence we could not ascertain exactly which component of this mixture or which interaction caused these organ dysfunctions.

## Conclusions

Herbal preparations may cause severe toxic effects, although they are often presented as natural products. In our case, ingestion of mixed herbal tea caused multiorgan toxicity including hepatotoxicity, bone marrow toxicity, and long-lasting nephrotoxicity. To reduce severe risks associated with the misuse of herbal products, it is very important to increase awareness among the general public and clinicians about the possible adverse effects of herbal preparations. Clinicians should consider the ingestion of herbal remedies as an etiology in the differential diagnosis of patients with unexplained organ dysfunction or toxic situations, and a detailed probing about herbal usage should be undertaken.
